# A Postmenopausal Woman with Giant Ovarian Serous Cyst Adenoma: A Case Report with Brief Literature Review

**DOI:** 10.1155/2018/5478328

**Published:** 2018-04-04

**Authors:** Nishat Fatema, Muna Mubarak Al Badi

**Affiliations:** Department of Gynaecology & Obstetric, Ibri Regional Hospital, Ministry of Health, Ibri, Oman

## Abstract

Giant (>10 cm) ovarian cyst is a rare finding. In the literature, a few cases of giant ovarian cysts have been mentioned sporadically, especially in elderly patients. We report a 57-year-old postmenopausal woman with a giant left ovarian cyst measuring 43 × 15 × 9 cm. She was referred to us from the local health center in view of palpable pelvic mass for six-month period. Considering the age and menopausal state, we performed a total abdominal hysterectomy and bilateral salpingo-oophorectomy with excision of the giant left ovarian cyst intact and successfully without any significant complication. On histopathological examination, the cyst was confirmed as benign serous cystadenoma of the ovary. During the management of these high-risk cases of multidisciplinary approach, intraoperative and postoperative strict vigilance is necessary to avoid unwanted complications.

## 1. Background

Giant ovarian tumor has become rare, because of the early detection of adnexal pathology with the advent of routine imaging modalities in the recent era of medical practice [1, 2].

In previous studies, the definition of large or giant ovarian cysts was described as cysts measuring more than 10 cm in diameter in the radiological scan or those cysts reaching above the umbilicus [1].

Cystadenoma, adenofibroma, and surface papillomas are the benign serous tumors. These tumors occur in about 25% of all benign ovarian neoplasms and 58% of all ovarian serous tumors [2].

Serous tumors are commonly seen during the reproductive period and 50% of them occur before the age of 40 years. Most of these cysts are benign in nature with the chance of malignancy being only 7%–13% in premenopausal and 8%–45% in postmenopausal women [3, 4].

Huge size ovarian serous cystadenoma is rare. In the literature, a few cases of giant ovarian cysts have been mentioned sporadically, primarily in elderly patients [2, 3].

We report a 57-year-old postmenopausal woman with a giant left ovarian cyst (43 × 15 × 9 cm). Considering the age and menopausal state, we performed the total abdominal hysterectomy (TAH) and bilateral salpingo-oophorectomy (BSO) with cystectomy for the patient. On histopathological examination, the cyst was confirmed as benign serous cyst adenoma of the ovary.

## 2. Case

A 57-year-old, para 04 postmenopausal woman was referred to our hospital from the local health center with a palpable pelvic mass for the last six months. She had been menopausal for the 7 years with the last childbirth occurring 17 years ago. She is a known case of hypothyroidism and on Tab. Thyroxin 50 microgram once daily. No significant surgical history was obtained.

On presentation, she was asymptomatic and had no complaints of anorexia, nausea vomiting, weight loss, or any postmenopausal bleeding. Her bowel and bladder habit was normal.

On general examination, she was found to be average built and weighing 62 kg. Abdominal examination revealed a pelvic mass extending beyond the umbilicus, corresponding to 26-week gravid uterus. The mass was mobile, firm, and nontender on palpation. On vaginal examination, cervix was found normal and fornixes were obliterated due to presence of the mass.

Laboratory tests were unremarkable except that the TFT value-TSH was 35.5 mIU/Li and free T4 was 6.9 pico mol/Li. Tumor markers were within normal limit, and CA 125 revealed 12 U/ml. Cervical PAP smear showed no evidence of dyskaryotic or malignant cells.

Radiological ultrasound revealed normal sized and shaped uterus with endometrial thickness 7 mm. A large left adnexal cyst was seen which was bilocular, thin smooth walled with clear anechoic contents measuring around 17.5 × 17.3 × 9.5 cm.

We did not conduct computerized tomography (CT) scans or magnetic resonance imaging (MRI) as the ultrasound scan findings were highly suggestive of a benign cyst, that is, a unilateral cyst with no solid areas or irregular surface and no ascites.

The calculated RMI (risk of malignancy index) was 1 × 3 × 12 = 36. Total score was USG score × menopausal score × Ca_125_ (U/Ml). USG score was as follows: 0, no risk factor; 1, one risk factor; 3, 2–5 risk factors. High-risk factors in USG were multilocular cysts, solid areas, bilateral lesions, ascites, and evidence of metastasis. Menopausal status was as follows: 1, premenopausal; 3, postmenopausal. Score < 200 indicates low risk (risk of ovarian malignancy is 0.15 times). Score > 200 indicates high risk (risk of ovarian malignancy is 42 times) [3].

We planned for TAH with BSO considering her age and menopausal status. After normalization of thyroid hormones value, we performed TAH with BSO.

**Figure 1:**
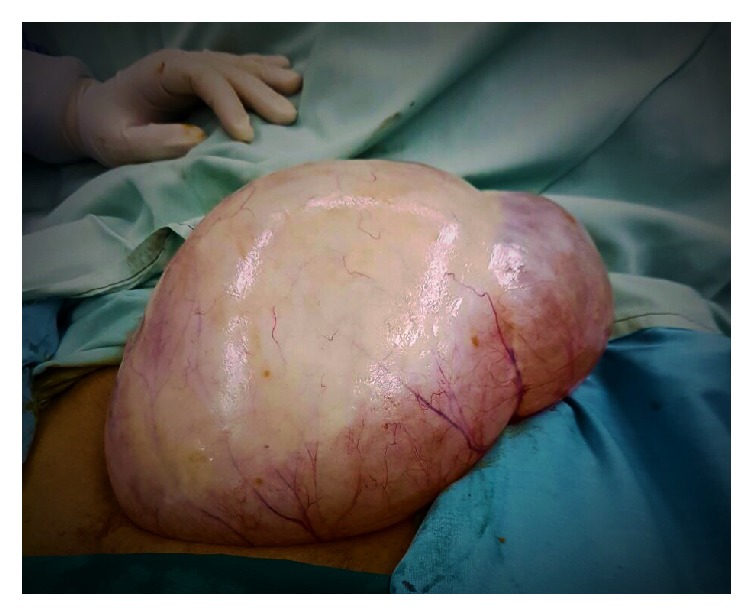
Left ovarian cyst (40 × 15 cm).

**Figure 2:**
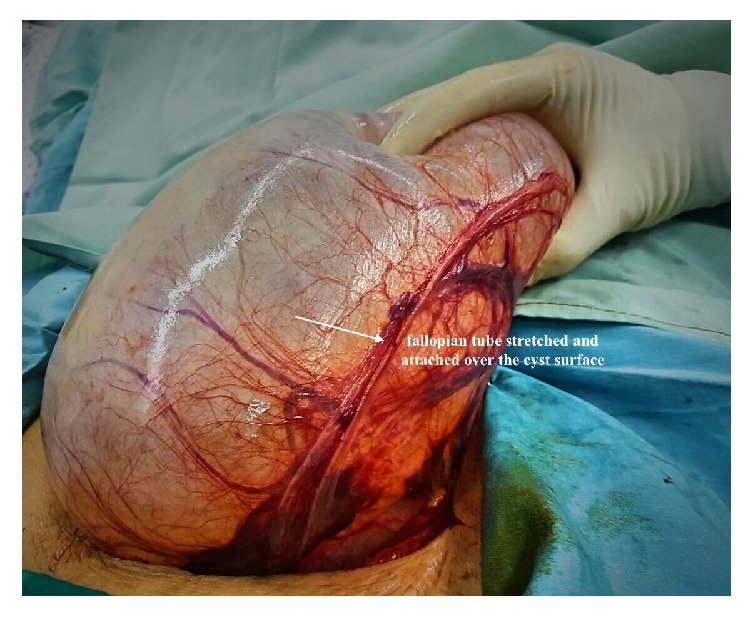
The left fallopian tube: adherent and stretched over the cyst.

**Figure 3:**
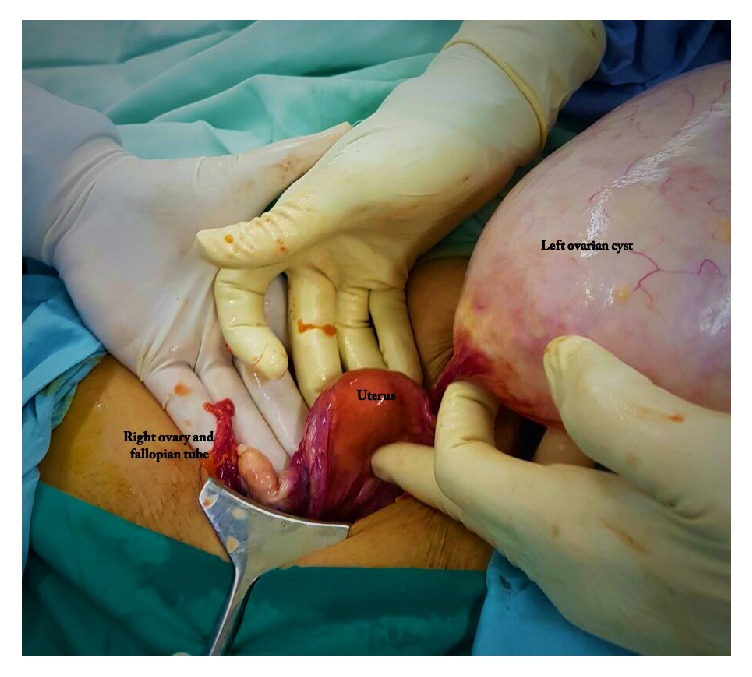
Healthy uterus, right fallopian tube, and ovary.

The abdomen was opened by a low transverse incision. Intraoperative around 40 × 15 cm sized left ovarian cyst (Figure 1) was seen; no healthy ovarian tissue was seen separately. The left tube was adherent and stretched over the cyst (Figure 2). Right tube, ovary, and the uterus were found healthy (Figure 3). There were no intraoperative complications and delay in total operative procedure. The blood loss was minimal.

On histopathology examination, the cyst was bilocular with smooth thin walled measuring 43 × 15 × 9 cm and lined by a single layer of bland flattened epithelial cells with occasional cuboidal epithelial cells. The cyst was filled with clear serous fluid. No malignant cells or nuclear atypia were observed. The histopathology was suggestive of benign serous cystadenoma of the ovary.

Her postoperative period was unremarkable. Oral feeding and ambulation were started 12 hours after the surgery. She was discharged on the fourth postoperative day in good condition.

## 3. Discussion

Large/giant ovarian cysts are benign in most of the cases and histopathologically these cysts are either serous or mucinous [4].

Serous tumors secrete serous fluids and are originated by invagination of the surface epithelium of ovary. Serous tumors are commonly benign (70%); 5–10% have borderline malignant potential, and 20–25% are malignant. Only 10% cases of all serous tumors are bilateral [3].

Serous cystadenomas are multilocular. In some instances, they include papillary projections. Giant ovarian serous cyst adenoma is a rare finding. In the literature, a few cases of giant ovarian cysts have been mentioned sporadically and especially in elderly postmenopausal women [2, 3].

Our presented case was a 57-year-old postmenopausal para 4 woman who experienced a palpable pelvic mass for six-month period without any other associated symptoms. We performed the total abdominal hysterectomy and bilateral salpingo-oophorectomy with the removal of an intact giant left ovarian serous cyst adenoma measuring 43 × 15 × 9 cm successfully.

Previous studies mentioned that the patients with huge adnexal mass are commonly presented with diffuse abdominal pain and distension, sometimes associated with anorexia and mechanical discomfort (Table 1). In this context, our patient had no significant symptoms except palpable pelvic mass for six-month period. In a reported case of mucinous cystadenoma, Madhu et al. mentioned that a patient was presented to them with the history of abdominal distention for the past 13 years and she sought medical management when her daily activity became restricted due to overdistension of abdomen [5]. Some other reported cases in previous studies were presented within a short period of time ranging from six months to two years, similar to our case (Table 1). In recent studies, the size of giant serous cyst adenoma of ovary was found in postmenopausal women measuring maximum 60 × 47 × 30 cm [2]. In most of the studies, tumor marker CA 125 was within normal range or mildly increased (Table 1). In this context, our patient's CA 125 level was also observed within the normal limit, 12 U/ml.

For the diagnosis of ovarian tumors, various imaging techniques are used. Pelvic ultrasonography, computed tomography, and magnetic resonance are the choice of imaging modalities that are used for the diagnosis of larger adnexal masses and metastatic involvement. Besides these, the serial measurements of the tumor marker CA-125 can be helpful [1].

We diagnosed the cyst by ultrasonography and the preoperative estimation of RMI (risk of malignancy index) to exclude malignancy. We did not conduct computerized tomography (CT) scans or magnetic resonance imaging (MRI), as the ultrasound scan findings were highly suggestive of a benign cyst, that is, a unilateral cyst with no solid areas or irregular surface and no ascites.

Large benign ovarian cysts are usually of two varieties—serous or mucinous—and because of their enlarged size and associated symptoms, they almost always require surgical intervention [4].

Extremely large ovarian cysts are traditionally managed by laparotomy. But the recent advances in endoscopic surgery have offered alternative choice by laparoscopic treatment of such extremely large ovarian cysts [8].

However, laparotomy and total excision of cysts are the choice of treatment in case of large ovarian cyst cases, until or unless prior to laparoscopic surgery ultrasound guided decompression or aspiration of the cyst is done [6].

As a first-line treatment modality for giant adnexal cysts, laparoscopy is still limited [9]. Due to technical difficulties, like restricted space, only a few surgeons practice laparoscopic management of extremely large ovarian cysts. In addition, there is a risk of cyst rupture and intra-abdominal spillage and trocar site implantation of malignant cells [4, 8].

In a review, Bellati and colleagues mentioned laparoscopically guided minilaparotomy (LGML) in case of benign large adnexal masses, with no other risk factor for malignancy, other than size. They concluded that in terms of safety and feasibility LGML is a better option in comparison to laparoscopy [10].

Excision of large ovarian cyst in women at reproductive age may damage ovarian reserve. In a literature review, the authors mentioned that bilateral cystectomies compared to unilateral cystectomy cause more damage to the ovarian reserve, but they observed recovery of ovarian reserve in both groups. Another study comparing unilateral cystectomy/ovariectomy with other abdominal and pelvic surgical interventions did not find any statistical difference in terms of long-term postoperative fertility [11, 12].

Regarding skin incision during excision of the large cysts, Madhu et al. noted that a low transverse incision associated with low risk of ventral hernia formation allows restoration of normal rectus abdominis muscle function. In contrast, vertical elliptical incision does not allow for adequate resection of the skin in the vertical plane [5, 13]. For our case, we opened the abdominal skin with a low transverse incision and successfully excised the cyst in intact condition without any complication.

Surgery is essential for large ovarian tumors even if benign [1]. Until now, there has been no randomized controlled trial for the laparoscopic management of ovarian cysts >20 cm, so laparotomy remained the ideal method for the excision of the giant ovarian cysts [4].

During surgical removal of large ovarian tumors, various intraoperative complications are reported in previous studies like splanchnic dilatation and venous pooling after the sudden removal of large intra-abdominal masses, and hypotension can occur due to decreased venous return resulting from obstructed inferior vena cava and pulmonary edema due to sudden reexpansion of a chronically collapsed lung, which occurred due to compression by the enlarged abdomen [1].

Hence, during the management of these high-risk cases of multidisciplinary approach, intraoperative and postoperative strict vigilance is necessary to avoid unwanted complications.

## Figures and Tables

**Table 1:** Giant ovarian cysts in postmenopausal women: literature review.

Study (year)	Age	Symptom	Site	Size of the cyst	Tumor marker CA 125 (U/ml)	Type of the cyst	Surgery
Sujatha and Babu (2009) [2]	66	Vague abdominal pain, anorexia	Unilateral	60 × 47 × 30 cm	46.61	Serous cyst adenoma	TAH + BSO

Alobaid et al. (2013) [4]	69	Abdominal distention and discomfort	Unilateral	Max diameter 20 cm	Normal	Serous cyst adenoma	LAVH + BSO

Madhu et al. (2013) [5]	55	Mechanical discomfort due to distended abdomen	Unilateral	50 × 39 × 47 cm	-* *-* *-	Mucinous cyst adenoma	TAH + BSO

Bhasin et al. (2017) [6]	85	Diffuse abdominal pain	Unilateral	58 × 46 cm	-* *-* *-	Mucinous cyst adenoma	Total excision of the cyst

Agrawal et al. 2015 [1]	65	Dull aching pain in the lower back, shortness of breath	Unilateral	25 × 28 × 15 cm	31.31	Serous cyst adenoma	TAH + BSO

Kim et al. 2016 [7]	52	Abdominal distention	Unilateral	36 × 21 × 30 cm	109.5I	Benign cystic lesion with hemorrhage	Total excision of the cyst
